# Histological features suggestive of survival in patients with renal cell carcinoma and tumor thrombus: A single-center experience

**DOI:** 10.3389/fonc.2022.980564

**Published:** 2022-09-05

**Authors:** Tao Wang, Yan Huang, Luojia Yang, Yang Yang, Di Li, Xiangyi Zhang, Xiaohui Ding, Baojun Wang, Xin Ma

**Affiliations:** ^1^ Medical School of Chinese PLA, Beijing, China; ^2^ Department of Urology, The Third Medical Centre, Chinese PLA General Hospital, Beijing, China; ^3^ Department of Pathology, The First Medical Centre, Chinese PLA General Hospital, Beijing, China

**Keywords:** tumor thrombus, histology, papillary renal cell carcinoma, follow-up, survival

## Abstract

**Purpose:**

To evaluate the impact of histological subtype on the survival of patients with renal cell carcinoma (RCC) and tumor thrombus (TT).

**Patients and methods:**

We retrospectively analyzed 350 patients with RCC and TT admitted to Chinese People’s Liberation Army General Hospital between January 2006 and June 2021. The patients underwent radical nephrectomy and thrombectomy using robot-assisted laparoscopic, laparoscopic, or open surgery. The clinical and pathological parameters of the patients were taken from their medical records. Survival was calculated with the Kaplan–Meier method. Univariate and multivariate Cox regression analyses were performed to evaluate the prognostic significance of variables on overall survival (OS) and progression-free survival (PFS).

**Results:**

TT levels 0–IV were observed in 132 (37.71%), 43 (12.29%), 134 (38.29%), 20 (5.71) and 21 (6.00%) patients, respectively. Papillary (pRCC), clear cell, and other histological subtypes of RCC were detected in 28 (8.00%), 286 (81.71%), and 36 (10.29%) patients, respectively. Compared to the clear cell cohort, collecting systemic invasion (46.43 vs. 25.17%; p = 0.030) and lymph node metastasis (39.29 vs. 11.54%; p < 0.01) were more common in the pRCC cohort. Kaplan–Meier analyses showed that patients with pRCC and other subtypes had significantly worse OS and PFS compared to patients with the clear cell subtype (p < 0.05). Multivariate analyses revealed that histology was independently associated with reduced OS and PFS, including among patients without lymph node and distant metastasis (N0M0).

**Conclusion:**

Papillary or other subtypes have a considerably shorter OS and PFS compared to clear cell subtype in RCC patients with TT. Strict follow-up and surveillance should be performed for papillary or other subtypes RCC with TT.

## Introduction

Renal cell carcinoma (RCC) often invades the renal venous system and causes tumor thrombus (TT), which affects 4–13% of newly diagnosed RCC patients and is associated with a poor prognosis (1). A higher grade and lymph node or distant metastasis are common at the time of RCC is diagnosed. Radical resection of TT significantly improves survival, and the 5-year survival rate of patients without metastasis is 40–65% (2–5).

The incidence of non–clear cell RCC (non-ccRCC) with TT is approximately 10% (4, 6, 7). Papillary RCC (pRCC) accounts for the largest proportion of non-ccRCC (8). Mancilla-Jimenez et al. (9) first described the features of pRCC in 1976. pRCC is classified into two subtypes based on cell and structure characteristics (10). Type II pRCC comprises large eosinophilic cells arranged in an irregular or pseudostratified manner, whereas type I RCC comprises small cuboidal cells with scant cytoplasm.

Several studies have evaluated prognostic factors in RCC patients with TT. However, only a few have compared ccRCC and pRCC, in addition to TT, which is largely because of the small sample size. Most previous studies divided RCC patients with TT into ccRCC and non-ccRCC groups. The histology of non-ccRCC has not been classified further, which has limited the significance of pathological stratification. In addition, pathological types other than ccRCC and pRCC with TT were often excluded by previous studies because of their rarity. Increasing attention has been paid to the study of rare renal pathological types associated with TT.

We summarized our 15-year experience with RCC and TT patients between 2006 and 2021. We classified cases into ccRCC, pRCC, and other histological type based on the histological findings. We evaluated the association between histological features of RCC and TT and prognosis.

## Materials and methods

### Study population and follow-up

After obtaining approval from our institutional review board, we retrospectively analyzed our renal tumor database and identified 350 patients with RCC and TT who underwent radical nephrectomy and complete thrombectomy between 2006 and 2021.

Clinical and pathological variables were recorded from the database. Before surgery, urinary system–enhanced computed tomography (CT) and/or magnetic resonance imaging (MRI) was performed to evaluate the diameter, location, and morphological characteristics of renal tumors; the presence or absence of lymph node metastasis; and length of IVC TT. Patients were evaluated before surgery for distant metastasis using imaging. Patients underwent robot-assisted laparoscopic, laparoscopic, or open surgery.

TT was classified according to the Mayo classification (11): level 0, renal vein; level I: <2 cm above renal vein; level II, infrahepatic: >2 cm but below intrahepatic vena cava; level III: intrahepatic portion of vena cava but below the diaphragm; and level IV, atrial: above diaphragm.

Pathology slides were reviewed retrospectively by a single pathologist and staged according to the TNM staging criteria of the American Joint Committee on Cancer (AJCC; 8th ed., 2017). The histological subtype (pRCC or ccRCC) was determined according to the 2004 WHO classification (12). The pRCC samples were subdivided into types I and II [10]. Grading was performed with the Fuhrman nuclear grading system (13).

### Measurement and outcomes

The patients were evaluated for postoperative recurrence and general conditions with blood chemistry and CT scan every 3 months for the first year. Thereafter, follow-up examinations were performed every 6 months. We constructed follow-up tables for patients using baseline information and data on survival time and survival status. Overall survival (OS) was defined as the time from first treatment to all-cause death or study end point. Progression-free survival (PFS) was defined as the time from first treatment to tumor progression or death.

### Statistical analysis

SPSS (v. 20.0; IBM, Armonk, NY, USA) was used for statistical analyses. Chi-square or Fisher’s exact tests were used for categorical variables, and one-way analysis of variance (ANOVA) was used for continuous variables. Two tailed p <0.05 was considered statistically significant. Kaplan-meier method was used to draw the survival curve. Univariable and multivariable Cox proportional hazards analyses were applied in the analysis of OS and PFS (“enter” algorithm). Variables achieving P value <0.05 in the univariate analysis were incorporated in the multivariable model to determine independent prognostic factors.

## Results

### Clinical and pathological characteristics

Of the 350 patients, 286 (81.71%), 28 (8.00%), and 36 (10.29%) had ccRCC, pRCC, and other histological type, respectively. The other types included chromophobe cell carcinoma, collecting duct carcinoma, unclassified RCC, neuroendocrine tumors, and mesenchymal tumors (Ewing sarcoma, leiomyosarcoma, synovial sarcoma, and angiomyolipoma). In the pRCC group, only one case had type 1; the remaining cases were type 2.

The mean age of all patients was 55.13 (12.56) years. According to the Mayo classification, TT levels 0–IV were present in 132 (37.71%), 43 (12.29%), 134 (38.29%), 20 (5.71%) and 21 (6.00%) patients, respectively. **Table 1** presents basic clinical and pathological information for the histological groups. Overall, 14.86% of patients had lymph node metastasis and 13.43% of them had distant metastasis.

**Table 1:** Comparison of clinical and pathological characteristics between clear cell renal cell carcinoma, papillary renal cell carcinoma and the other subtype.

	overall (n=350)	ccRCC (n=286)	pRCC (n=28)	others (n=36)	P value
**Age, yr , n (%)**					
≤60	221 (63.14)	175 (61.19)	20 (71.43)	26 (72.22)	0.277
>60	129 (36.86)	111 (38.81)	8 (28.57)	10 (27.78)	
Sex, n (%) *					
Male	255 (72.86)	223 (77.97)	17 (60.71)	15 (41.67)	<0.001*
Female	95 (27.14)	63 (22.03)	11 (39.29)	21 (58.33)	
BMI, n (%) *					
≤25	187 (53.43)	142 (49.65)	19 (67.86)	26 (72.22)	0.011*
>25	163 (46.57)	144 (50.35)	9 (32.14)	10 (27.78)	
Mean (SD)	24.72 (3.64)	25.01 (3.62)	23.65 (3.63)	23.28 (3.39)	0.007
**Laterality, n (%)**					
Left	139 (39.71)	116 (40.56)	13 (46.43)	10 (27.78)	0.252
Right	211 (60.29)	170 (50.44)	15 (53.57)	26 (72.22)	
**Preoperative** **hematuria, n (%)**					
No	212 (60.57)	173 (60.49)	13 (46.43)	26 (72.22)	0.111
Yes	138 (39.43)	113 (39.51)	15 (53.57)	10 (27.78)	
**Hypertension, n (%)**					
No	233 (66.57)	183 (63.99)	22 (78.57)	28 (77.78)	0.095
Yes	117 (33.43)	103 (36.01)	6 (21.43)	8 (22.22)	
**Diabetes, n (%)**					
No	283 (80.86)	228 (79.72)	26 (92.86)	29 (80.56)	0.241
Yes	67 (19.14)	58 (20.28)	2 (7.14)	7 (19.44)	
**Surgical approach,** **n (%)**					
Open	108 (30.86)	92 (32.17)	7 (25.00)	9 (25.00)	0.244
Laparoscopy	49 (14.00)	44 (15.28)	3 (10.71)	2 (5.56)	
Robot-assisted	193 (55.14)	150 (52.45)	18 (64.29)	25 (69.44)	
**Surgery time (min)**	215.81 (124.85)	213.15 (128.51)	222.86 (126.53)	231.33 (90.95)	0.680
**Bleeding (ml)**	999.08 (1450.44)	1009.65 (1477.42)	1246.43 (1723.20)	723.06 (887.05)	0.345
**Tumor size (cm)**					
≤7	164 (46.86)	139 (48.60)	12 (42.86)	13 (36.11)	0.333
>7	186 (53.14)	147 (51.40)	16 (57.14)	23 (63.89)	
Mean (SD)	7.98 (3.25)	7.83 (3.12)	8.55 (3.45)	8.78 (3.97)	0.158
**T stage, n (%)**					
T3a	120 (34.29)	104 (36.36)	9 (32.14)	7 (19.44)	0.143
T3b	166 (47.43)	129 (45.10)	13 (46.43)	24 (66.67)	
T3c	44 (12.57)	39 (13.64)	3 (10.71)	2 (5.56)	
T4	20 (5.71)	14 (4.90)	3 (10.71)	3 (8.33)	
N status*					
N0	298 (85.14)	253 (88.46)	17 (60.71)	28 (77.78)	<0.001*
N1	52 (14.86)	33 (11.54)	11 (39.29)	8 (22.22)	
**Metastasis, n (%)**					
M0	303 (86.57)	247 (86.36)	22 (78.57)	34 (94.44)	0.176
M1	47 (13.43)	39 (13.64)	6 (21.43)	2 (5.56)	
**TT level (Mayo), n (%)**					
0	132 (37.71)	113 (39.51)	11 (39.29)	8 (22.22)	0.202
I	43 (12.29)	39 (13.64)	2 (7.14)	2 (5.56)	
II	134 (38.29)	100 (34.97)	13 (46.43)	21 (58.33)	
III	20 (5.71)	16 (5.59)	1 (3.57)	3 (8.33)	
IV	21 (6.00)	18 (6.29)	1 (3.57)	2 (5.56)	
**Grade/Fuhrman, n (%)**					
1+2	135 (43.97)	125 (46.13)	7 (33.33)	3 (20.00)	0.083
3+4	172 (56.03)	146 (53.87)	14 (66.67)	12 (80.00)	
**Length of IVC TT,** **n (%)**					
≤5	121 (56.28)	97 (56.73)	8 (47.06)	16 (59.26)	0.452
>5	94 (43.72)	74 (43.27)	9 (52.94)	11 (40.74)	
Mean (SD)	5.40 (3.35)	5.42 (3.47)	5.01 (2.38)	5.48 (3.15)	0.882
Sarcomatoid differentiation, n (%) *					
No	331 (94.57)	276 (96.50)	26 (92.86)	29 (80.56)	<0.001*
Yes	19 (5.43)	10 (3.50)	2 (7.14)	7 (19.44)	
**Necrosis, n (%)**					
No	165 (47.14)	132 (46.15)	14 (50.00)	19 (52.78)	0.718
Yes	185 (52.86)	154 (53.85)	14 (50.00)	17 (47.22)	
**Fat invasion, n (%)**					
No	282 (80.57)	235 (82.17)	19 (67.86)	28 (77.78)	0.171
Yes	68 (19.43)	51 (17.83)	9 (32.14)	8 (22.22)	
**Sinus fat invasion,** **n (%)**					
No	232 (66.29)	191 (66.78)	15 (53.57)	26 (72.22)	0.269
Yes	118 (33.71)	95 (33.22)	13 (46.43)	10 (27.78)	
Collecting system invasion, n (%)*					
No	252 (72.00)	214 (74.83)	15 (53.57)	23 (63.89)	0.030*
Yes	98 (28.00)	72 (25.17)	13 (46.43)	13 (36.11)	

ccRCC, clear cell renal cell carcinoma; pRCC, papillary renal cell carcinoma; TT, tumor thrombus; IVC, inferior vein cava.

*Means P < 0.05.

The proportions of high body mass index (BMI; 50.35 vs. 27.78 kg/m2; p = 0.011) and male sex (77.97 vs. 41.67%; p < 0.01) were higher in the ccRCC group than the other histological type group. The proportion of sarcomatoid differentiation was significantly higher in the other histological type group than the ccRCC group (19.44 vs. 3.50%; p < 0.01). The proportions of collecting system invasion (46.43 vs. 25.17%; p = 0.030) and lymph node metastasis (39.29 vs. 11.54%; p < 0.01) were significantly higher in the pRCC group than the ccRCC group. There were no significant differences among the groups in terms of age, laterality, preoperative hematuria, hypertension, diabetes, surgical approach, surgery time, bleeding, tumor size, T stage, distant metastasis, TT level, grade, length of IVC TT, necrosis, fat invasion, or sinus fat invasion.

### Prognosis

**Figure 1:**
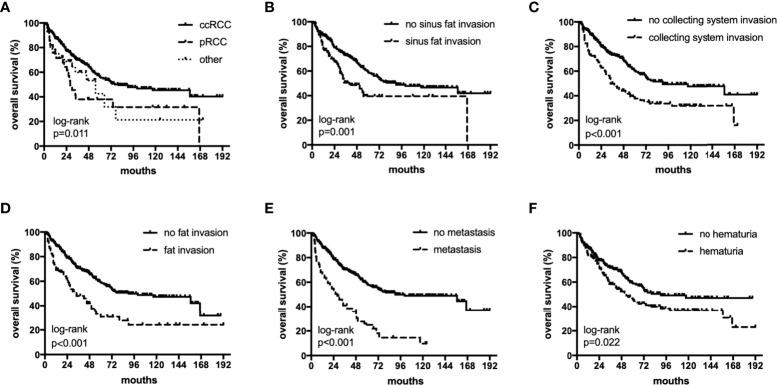
The subgroup OS rates of histology **(A)**, sinus fat invasion **(B)**, perioperative hematuria **(C)**, collecting system invasion **(D)**, fat invasion **(E)**, and distant metastasis **(F)**.

**Figure 2:**
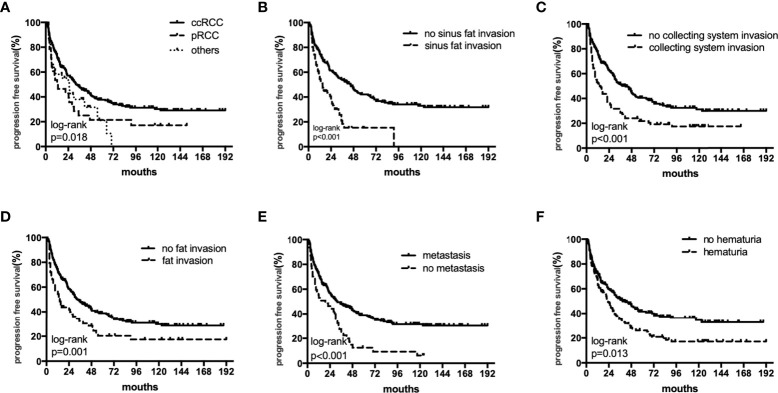
The subgroup PFS rates of histology **(A)**, sinus fat invasion **(B)**, perioperative hematuria **(C)**, collecting system invasion **(D)**, fat invasion **(E)**, and distant metastasis **(F)**.

The median OS were 40 (18–82), 27.5 (12–55) and 36 (17–75) months,respectively. The median PFS were 25 (11–64), 17(6-55) and 11(5-26) months,respectively. In univariate analyses, BMI, histology, preoperative hematuria, tumor size, N status, distant metastasis, grade, sarcomatoid differentiation, necrosis, fat invasion, sinus fat invasion, and collecting system invasion were significant factors that affected OS (p < 0.05; **Figure 1**, **Table 2**). In comparison, histology, preoperative hematuria, tumor size, distant metastasis, grade, necrosis, fat invasion, sinus fat invasion, and collecting system invasion were significant factors that affected PFS (p < 0.05; **Figure 2**, **Supplementary Table 1**). The differences in OS and PFS among histological types remained significant when analyses were restricted to N0M0 patients (**Supplementary Tables 2, 3**).

**Table 2:** Univariable and multivariate Cox proportional hazards regression for overall survival.

	Univariate		Multivariate	
	HR (95%CI)	P value	HR (95%CI)	P value
**Age (>60 vs. ≤60)**	1.007 (0.728 - 1.394)	0.966		
**Age (continue)**	0.996 (0.983 - 1.008)	0.512		
**Sex (female vs. male)**				
**BMI (>25 vs. ≤25)**	0.675 (0.491 - 0.927)	0.015	0.726 (0.507 - 1.040)	0.081
**BMI (continue)**	0.945 (0.904 - 0.988)	0.012		
**Laterality (right vs. left)**	0.845 (0.617 - 1.156)	0.291		
**Preoperative hematuria** **(yes vs. no)**	1.438 (1.052 - 1.967)	0.023	1.774 (1.247 - 2.525)	0.001
**Hypertension (yes vs. no)**	1.026 (0.734-1.434)	0.881		
**Diabetes (yes vs. no)**	1.316 (0.903 - 1.917)	0.153		
**Surgical approach**				
open	Referent			
laparoscopy	0.763 (0.481 - 1.209)	0.249		
robot-assisted	0.801 (0.565 - 1.136)	0.212		
**Surgery time**	1.001 (1.000 - 1.002)	0.219		
**Bleeding**	1.000 (1.000 - 1.000)	0.475		
**Histology**				
ccRCC	Referent			
pRCC	1.914 (1.179 - 3.107)	0.009	1.579 (0.635-2.187)	0.026
other	1.565 (0.940 - 2.608)	0.025	2.473 (1.184 - 5.164)	0.016
**Tumor size (>7 vs. ≤7)**	1.490 (1.081 - 2.052)	0.015	1.135 (0.782 - 1.648)	0.504
**Tumor size (continue)**	1.077 (1.028 - 1.129)	0.002		
**T stage**				
T3a	Referent			
T3b	0.945 (0.669 - 1.335)	0.749		
T3c	1.120 (0.644 - 1.946)	0.688		
T4	1.733 (0.913 - 3.289)	0.093		
**N status (N1 vs. N0)**	1.665 (1.127 - 2.460)	0.010	1.073 (0.667 - 1.727)	0.771
**Metastasis (M1 vs. M0)**	2.873 (1.983 - 4.162)	<0.001	3.000 (1.964 - 4.582)	<0.001
**TT level (Mayo)**				
I	Referent			
II	1.037 (0.732 - 1.469)	0.838		
III	1.068 (0.517 - 2.206)	0.859		
IV	1.081 (0.523 - 2.233)	0.834		
**Grade/Furhman (3+4 vs. 1+2)**	1.864 (1.305 - 2.662)	0.001	1.341 (0.916 - 1.963)	0.131
**Length of IVC TT (>5 vs. ≤5)**	1.029 (0.968-1.095)	0.360		
**Length of IVC TT (continue)**	1.181 (0.766 - 1.822)	0.452		
**Sarcomatoid differentiation** **(yes vs. no)**	1.994 (1.128 - 3.525)	0.018	1.032 (0.519 - 2.052)	0.929
**Necrosis (yes vs. no)**	1.530 (1.114 - 2.102)	0.009	1.351 (0.938 - 1.947)	0.106
**Fat invasion (yes vs. no)**	2.071 (1.457 - 2.944)	<0.001	1.956 (1.286 - 2.974)	0.002
**Sinus fat invasion** **(yes vs. no)**	1.778 (1.263 - 2.502)	0.001	1.787 (1.250 - 2.554)	0.001
**Collecting system invasion (yes vs. no)**	1.866 (1.357 - 2.567)	<0.001	1.543 (1.025 - 2.324)	0.038

ccRCC, clear cell renal cell carcinoma; pRCC, papillary renal cell carcinoma; TT, tumor thrombus; CI, confidence interval; HR, hazard ratio; IVC, inferior vein cava.

In multivariate analyses, histology was an independent predictor of OS and PFS (p < 0.05). In addition, perioperative hematuria, distant metastasis, fat invasion, sinus fat invasion, and collecting system invasion were independent risk factors for OS and PFS (**Table 2**, **Supplementary Table 1**). In multivariate analyses, when analyses were restricted to N0M0 patients, histology remained significantly associated with OS and PFS (**Supplementary Tables 2, 3**).

The 5-year OS rates in the ccRCC, pRCC, and other histological type groups were 56.0%, 33.5%, and 33.3%, respectively. The 5-year PFS rates in the ccRCC, pRCC, and other histological type groups were 37.7%, 28.0%, and 27.3%, respectively (**Table 3**). In summary, the combination of pRCC or other histological type with TT indicated a worse prognosis than ccRCC with TT.

**Table 3:** Survival comparison of the three groups.

	1 year survival rate		3 year survival rate		5 year survival rate	
	OS	PFS	OS	PFS	OS	PFS
**ccRCC**	87.7%	73.4%	70.0%	48.0%	56.0%	37.7%
**pRCC**	75.9%	58.6%	43.8%	41.1%	33.5%	28.0%
**other**	73.0%	56.6%	58.2%	44.8%	33.3%	27.3%

ccRCC, clear cell renal cell carcinoma; pRCC, papillary renal cell carcinoma; OS, overall survival; PFS, progression free survival.

## Discussion

There is no accepted consensus on the prognostic significance of histological classification of RCC with TT. On the one hand, the sample size is small and the persuasiveness is relatively poor, and on the other hand, the previous literature is contradictory and controversial. Therefore, as the largest TT diagnosis and treatment center in China, we conducted research to make up for this shortage.

We examined the outcomes of 350 RCC patients with TT. The 5-year OS rate for RCC patients with TT was 53.4%, which is consistent with previous studies that have shown 5-year survival rates of 40–65% (2, 3, 5). Our study cases had ccRCC (82%) and pRCC (8%) variants. The incidence of pRCC is similar in the present study and previous studies (6).

Although prognostic factors for RCC patients with TT are well established, the effects of histology on prognosis are controversial. Margulis et al. (14) reported that TT was an independent prognostic marker in pRCC patients but not ccRCC patients. Mancilla et al. (9) reported that venous TT resulted in poor survival in pRCC patients. Kim et al. (15) reported that type II papillary histology predicted a poor outcome in RCC patients with vena cava TT. Ciancio et al. (16) identified non–clear cell histology with TT as independent prognostic factors for poor disease-specific survival. In the largest multicenter study reported to date (1,774 cases), Tilki et al. (17) found that pRCC patients with vena cava TT had significantly worse cancer-specific outcomes compared to patients with other RCC histological subtypes; these findings are consistent with our results.

However, some studies have drawn different conclusions. Terakawa et al. (18) and Wagner et al. (5) reported that histological subtype (ccRCC vs. others) in TT was a significant prognostic predictor in univariate analyses but not in multivariate analyses. Kaushik et al. (19) found that non-ccRCC patients with TT did not experience greater disease recurrence or worse survival compared to ccRCC patients. In our study, pRCC and other histological subtypes with TT were significant factors in multivariate analyses, and the ccRCC subtype was associated with superior outcomes.

Steffens et al. (20) found that pRCC patients had a significantly better prognosis, and an advanced subgroup had a worse prognosis, compared to ccRCC patients. These conflicting results for the effects of papillary histology might be due to different ratios of pRCC types 1 and 2. Type 2 pRCC is more aggressive and has a worse outcome than type 1 pRCC (21, 22). Two previous studies (23, 24) enrolled 7 and 25 type 2 pRCC patients with TT and found a worse prognosis compared to ccRCC patients. Of the 28 cases of pRCC with TT included in the present study, 27 had type 2 pRCC and only 1 had type 1, which partly explains the poor prognosis in the pRCC group. We found that the pRCC subtype had more frequent lymph node metastasis and collecting system invasion compared to ccRCC, which may reflect the malignant potential of pRCC with TT (25, 26). The poor survival outcomes of pRCC patients with TT might be due in part to a lack of effective treatment for this subtype (14), which suggests a need to develop effective treatments. In addition, we separately analyzed the N0M0 subgroup and found that histology remained a significant factor.

In terms of surgical methods, 52.45% of patients in the present study underwent robot-assisted laparoscopic surgery. In previous studies, most patients underwent open or laparoscopic surgery. In recent years, with the popularization of and improvements in novel surgery technology, an increasing number of patients have been selecting more minimally invasive approaches. Therefore, the results of our study are more instructive. Note that we did not find a difference in patient outcomes by surgical method.

Several limitations of the study need to be acknowledged. First, this was a single-center retrospective analysis with the limitations inherent to this study type and possible uncontrolled confounding factors. Second, the fact that the surgeries were not performed by the same surgeon may have introduced bias into the study results. However, our study results are more generalizable because multiple surgeons performed the surgeries, similar to real-world practice. Despite these limitations, to the best of our knowledge, this is the largest single-center study to use multivariate analysis to evaluate the prognostic effects of histological subtype in RCC patients with TT.

## Conclusion

Early recognition of patients who are at high risk for mortality is essential. Previous studies have had small sample sizes, whereas the present single-center study retrospectively analyzed 28 cases of pRCC and 286 cases of ccRCC with TT. We found that histological subtype was a significant independent prognostic factor, with significantly worse OS and PFS for pRCC and other histological type compared to ccRCC. Therefore, aggressive renal hilar lymphadenectomy and targeted and adjuvant therapy clinical trials are required to reduce postoperative recurrence and improve oncological outcomes in pRCC and other histological type with TT. Active postoperative surveillance and close follow-up are also required for those patients.

## Data availability statement

The original contributions presented in the study are included in the article/**Supplementary Material**. Further inquiries can be directed to the corresponding authors.

## Ethics statement

The studies involving human participants were reviewed and approved by Chinese PLA General Hospital. The patients/participants provided their written informed consent to participate in this study.

## Author contributions

Conception and design: YH and TW. Acquisition of data: LY. Analysis and interpretation of data: YY and DL. Writing, review, and/or revision of the manuscript: TW, YY, XZ. Administrative, technical, or material support: BW and XM. Study supervision: XD. All authors contributed to the article and approved the submitted version.

## Funding

This work was financially supported by Hospital Level Project of PLA General Hospital of China (No.2020-JQPY-002).

## Conflict of interest

The authors declare that the research was conducted in the absence of any commercial or financial relationships that could be construed as a potential conflict of interest.

## Publisher’s note

All claims expressed in this article are solely those of the authors and do not necessarily represent those of their affiliated organizations, or those of the publisher, the editors and the reviewers. Any product that may be evaluated in this article, or claim that may be made by its manufacturer, is not guaranteed or endorsed by the publisher.
