# Supplementary material for: Histological features suggestive of survival in patients with renal cell carcinoma and tumor thrombus: A single-center experience
Source: Front Oncol. 2022 Sep 5;12:980564. doi: 10.3389/fonc.2022.980564 (PMC9483090; doi:10.3389/fonc.2022.980564)
Supplement: Supplementary file 1 [file DataSheet_1.docx]

**Supplementary table1 |** Univariable and multivariate Cox proportional hazards regression for progression free survival.

|  | Univariate | | Multivariate | |
| --- | --- | --- | --- | --- |
|  | HR (95%CI) | P value | HR (95%CI) | P value |
| **Age(>60 vs. ≤60)** | 0.958(0.727-1.263) | 0.762 |  |  |
| **Age(continue)** | 0.996(0.985-1.007) | 0.457 |  |  |
| **Sex(female vs. male)** | 0.946(0.696-1.287) | 0.726 |  |  |
| **BMI(>25 vs. ≤25)** | 0.768(0.588-1.004) | 0.054 |  |  |
| **BMI(continue)** | 0.969(0.933-1.005) | 0.091 |  |  |
| **Laterality(right vs. left)** | 0.815(0.624-1.065) | 0.134 |  |  |
| **Preoperative hematuria**  **(yes vs. no)** | 1.538(1.179-2.008) | 0.002 | 1.701(1.266-2.285) | <0.001 |
| **Hypertension**  **(yes vs. no)** | 1.077(0.814-1.424) | 0.605 |  |  |
| **Diabetes(yes vs. no)** | 1.128(0.810-1.571) | 0.474 |  |  |
| **Surgical approach** |  |  |  |  |
| open | Referent |  |  |  |
| laparoscopy | 1.167(0.780-1.746) | 0.452 |  |  |
| robot-assisted | 1.300(0.957-1.765) | 0.093 |  |  |
| **Surgery time** | 1.001(1.000-1.002) | 0.034 |  |  |
| **Bleeding** | 1.000(1.000-1.000) | 0.901 |  |  |
| **Histology** |  |  |  |  |
| ccRCC | Referent |  |  |  |
| pRCC | 2.255(1.437-3.539) | 0.028 | 1.247(0.730-2.133) | 0.041 |
| other | 1.547(1.006-2.379) | 0.047 | 1.719(0.919-3.215) | 0.009 |
| **Tumor size(>7 vs. ≤7)** | 1.369(1.046-1.792) | 0.022 | 1.198(0.883-1.626) | 0.245 |
| **Tumor size(continue)** | 1.048(1.007-1.091) | 0.022 |  |  |
| **T stage** |  |  |  |  |
| T3a | Referent |  |  |  |
| T3b | 1.107(0.823-1.488) | 0.502 |  |  |
| T3c | 1.489(0.959-2.313) | 0.076 |  |  |
| T4 | 1.364(0.758-2.454) | 0.300 |  |  |
| **N status(N1 vs. N0)** | 1.407(0.991-1.998) | 0.056 |  |  |
| **Metastasis(M1 vs. M0)** | 1.977(1.405-2.782) | <0.001 | 1.825(1.259-2.646) | 0.001 |
| **TT level (Mayo)** |  |  |  |  |
| I | Referent |  |  |  |
| II | 1.093(0.815-1.466) | 0.553 |  |  |
| III | 1.542(0.900-2.643) | 0.115 |  |  |
| IV | 1.195(0.659-2.167) | 0.558 |  |  |
| **Grade/Furhman**  **(3+4 vs. 1+2)** | 1.783(1.319-2.411) | <0.001 | 1.316(0.948-1.828) | 0.101 |
| **Length of IVC TT**  **(>5 vs. ≤5)** | 1.127(0.791-1.607) | 0.508 |  |  |
| **Length of IVC TT (continue)** | 1.019(0.968-1.074) | 0.465 |  |  |
| **Sarcomatoid differentiation**  **(yes vs. no)** | 1.391(0.809-2.391) | 0.233 |  |  |
| **Necrosis(yes vs. no)** | 1.422(1.088-1.859) | 0.010 | 1.257(0.923-1.714) | 0.147 |
| **Fat invasion(yes vs. no)** | 1.696(1.238-2.321) | 0.001 | 1.517(1.065-2.160) | 0.021 |
| **Sinus fat invasion**  **(yes vs. no)** | 1.833(1.380-2.434) | <0.001 | 1.605(1.180-2.183) | 0.003 |
| **Collecting system invasion(yes vs. no)** | 1.718(1.300-2.271) | <0.001 | 1.597(1.149-2.220) | 0.005 |

Note: ccRCC = clear cell renal cell carcinoma; pRCC = papillary renal cell carcinoma; TT = tumor thrombus; IVC = inferior vein cava.

**Supplementary table2 |** Univariable and multivariate Cox proportional hazards regression for overall survival:N0M0 subgroup.

|  | **Univariate** | | **Multivariate** | |
| --- | --- | --- | --- | --- |
|  | **HR (95%CI)** | **P value** | **HR (95%CI)** | **P value** |
| **Age(>60 vs. ≤60)** | 1.077(0.717-1.617) | 0.721 |  |  |
| **Age(continue)** | 0.999(0.982-1.015) | 0.866 |  |  |
| **Sex(female vs. male)** | 1.303(0.852-1.992) | 0.222 |  |  |
| **BMI(>25 vs. ≤25)** | 0.651(0.437-0.970) | 0.035 | 0.542(0.335-0.878) | 0.013 |
| **BMI(continue)** | 0.920(0.870-0.972) | 0.003 |  |  |
| **Laterality(right vs. left)** | 0.942(0.631-1.406) | 0.768 |  |  |
| **Preoperative hematuria**  **(yes vs. no)** |  |  |  |  |
| **Hypertension(yes vs. no)** | Referent |  |  |  |
| **Diabetes(yes vs. no)** | 2.395(1.199-4.785) | 0.013 | 1.277(0.565-2.889) | 0.037 |
| **Surgical approach** | 1.616(0.855-3.054) | 0.014 | 1.667(0.597-4.657) | 0.029 |
| open |  |  |  |  |
| laparoscopy | Referent |  |  |  |
| robot-assisted | 1.096(0.712-1.689) | 0.677 |  |  |
| **Surgery time** | 1.371(0.546-3.443) | 0.502 |  |  |
| **Bleeding** | 1.318(0.564-3.077) | 0.524 |  |  |
| **Histology** | 0.920(0.597-1.418) | 0.706 |  |  |
| ccRCC | 1.661(1.069-2.582) | 0.024 | 2.390(1.434-3.984) | 0.001 |
| pRCC | 1.370(0.924-2.032) | 0.117 |  |  |
| other | 1.067(1.003-1.134) | 0.039 | 1.001(0.927-1.080) | 0.980 |
| **Tumor size(>7 vs. ≤7)** | 1.799(1.218-2.659) | 0.003 | 1.512(0.977-2.340) | 0.063 |
| **Tumor size(continue)** | 1.539(1.036-2.285) | 0.033 | 1.171(0.747-1.837) | 0.490 |
| **T stage** | 2.195(1.014-4.749) | 0.046 | 1.092(0.422-2.830) | 0.856 |
| T3a |  |  |  |  |
| T3b | Referent |  |  |  |
| T3c | 0.751(0.434-1.301) | 0.307 |  |  |
| T4 | 0.764(0.490-1.192) | 0.236 |  |  |
| **N status(N1 vs. N0)** |  |  |  |  |
| **Metastasis(M1 vs. M0)** | Referent |  |  |  |
| **TT level (Mayo)** | 1.029(0.674-1.569) | 0.896 |  |  |
| I | 1.345(0.699-2.588) | 0.375 |  |  |
| II | 1.864(0.250-13.898) | 0.544 |  |  |
| III | 2.421(1.562-3.751) | 0.000 | 2.072(1.222-3.514) | 0.007 |
| IV | 2.314(1.495-3.580) | 0.000 | 1.534(0.944-2.491) | 0.084 |
| **Grade/Furhman(3+4 vs. 1+2)** | 1.530(0.908-2.578) | 0.110 |  |  |
| **Length of IVC TT(>5 vs. ≤5)** | 1.073(0.995-1.158) | 0.068 |  |  |
| **Length of IVC TT (continue)** | 1.001(0.999-1.003) | 0.187 |  |  |
| **Sarcomatoid differentiation**  **(yes vs. no)** | 1.000(1.000-1.000) | 0.011 | 1.000(1.000-1.000) | 0.340 |
| **Necrosis(yes vs. no)** | 2.369(1.533-3.662) | 0.000 | 1.652(1.028-2.656) | 0.027 |
| **Fat invasion(yes vs. no)** | 2.208(1.484-3.285) | 0.000 | 1.826(1.072-3.107) | 0.038 |

Note: ccRCC = clear cell renal cell carcinoma; pRCC = papillary renal cell carcinoma; TT = tumor thrombus; IVC = inferior vein cava.

**Supplementary table3 |** Univariable and multivariate Cox proportional hazards regression for progression free survival: N0M0 subgroup.

|  | **Univariate** | | **Multivariate** | |
| --- | --- | --- | --- | --- |
|  | **HR (95%CI)** | **P value** | **HR (95%CI)** | **P value** |
| **Age(>60 vs. ≤60)** | 0.939(0.677-1.302) | 0.704 |  |  |
| **Age(continue)** | 0.996(0.983-1.009) | 0.543 |  |  |
| **Sex(female vs. male)** | 0.974(0.686-1.383) | 0.882 |  |  |
| **BMI(>25 vs. ≤25)** | 0.757(0.553-1.038) | 0.084 |  |  |
| **BMI(continue)** | 0.965(0.924-1.009) | 0.116 |  |  |
| **Laterality(right vs. left)** | 0.807(0.586-1.111) | 0.188 |  |  |
| **Preoperative hematuria**  **(yes vs. no)** |  |  |  |  |
| **Hypertension(yes vs. no)** | Referent |  |  |  |
| **Diabetes(yes vs. no)** | 2.123(1.173-3.843) | 0.013 | 1.265(0.614-2.606) | 0.045 |
| **Surgical approach** | 1.457(0.885-2.398) | 0.039 | 2.127(0.946-4.783) | 0.028 |
| open |  |  |  |  |
| laparoscopy | Referent |  |  |  |
| robot-assisted | 1.136(0.804-1.605) | 0.471 | 0.721(0.420-1.239) | 0.236 |
| **Surgery time** | 2.361(1.306-4.270) | 0.004 | 1.824(0.705-4.720) | 0.215 |
| **Bleeding** | 1.682(0.890-3.177) | 0.109 | 1.206(0.395-3.681) | 0.742 |
| **Histology** | 1.104(0.792-1.539) | 0.560 |  |  |
| ccRCC | 1.237(0.846-1.808) | 0.273 |  |  |
| pRCC | 1.492(1.089-2.043) | 0.013 | 1.085(0.752-1.566) | 0.664 |
| other | 1.058(1.007-1.111) | 0.025 |  |  |
| **Tumor size(>7 vs. ≤7)** | 1.711(1.251-2.340) | 0.001 | 1.470(1.023-2.113) | 0.037 |
| **Tumor size(continue)** | 1.638(1.195-2.247) | 0.002 | 1.435(0.999-2.061) | 0.051 |
| **T stage** | 1.393(0.683-2.838) | 0.362 |  |  |
| T3a |  |  |  |  |
| T3b | Referent |  |  |  |
| T3c | 1.135(0.706-1.825) | 0.602 |  |  |
| T4 | 1.334(0.913-1.947) | 0.136 |  |  |
| **N status(N1 vs. N0)** |  |  |  |  |
| **Metastasis(M1 vs. M0)** | Referent |  |  |  |
| **TT level (Mayo)** | 1.199(0.849-1.693) | 0.302 | 1.123(0.658-1.917) | 0.671 |
| I | 1.775(1.082-2.913) | 0.023 | 0.875(0.322-2.378) | 0.793 |
| II | 0.652(0.090-4.745) | 0.673 | 0.149(0.016-1.412) | 0.097 |
| III | 1.938(1.334-2.815) | 0.001 | 1.746(1.099-2.773) | 0.018 |
| IV | 2.342(1.642-3.341) | 0.000 | 1.707(1.147-2.540) | 0.008 |
| **Grade/Furhman(3+4 vs. 1+2)** | 1.445(0.971-2.151) | 0.070 |  |  |
| **Length of IVC TT(>5 vs. ≤5)** | 1.050(0.998-1.116) | 0.119 |  |  |
| **Length of IVC TT (continue)** | 1.002(1.001-1.003) | 0.001 | 1.001(0.999-1.003) | 0.441 |
| **Sarcomatoid differentiation**  **(yes vs. no)** | 1.000(1.000-1.000) | 0.002 | 1.000(1.000-1.000) | 0.479 |
| **Necrosis(yes vs. no)** | 2.202(1.574-3.080) | 0.000 | 1.471(0.938-2.307) | 0.093 |
| **Fat invasion(yes vs. no)** | 1.989(1.428-2.771) | 0.000 | 1.600(1.076-2.379) | 0.020 |

Note: ccRCC = clear cell renal cell carcinoma; pRCC = papillary renal cell carcinoma; TT = tumor thrombus; IVC = inferior vein cava.
